# Analyses of Sporocarps, Morphotyped Ectomycorrhizae, Environmental ITS and LSU Sequences Identify Common Genera that Occur at a Periglacial Site

**DOI:** 10.3390/jof1010076

**Published:** 2015-05-25

**Authors:** Ari Jumpponen, Shawn P. Brown, James M. Trappe, Efrén Cázares, Rauni Strömmer

**Affiliations:** 1 Division of Biology, Kansas State University, Manhattan, KS 66506, USA; 2 Ecological Genomics Institute, Kansas State University, Manhattan, KS 66506, USA; 3 Department of Plant Biology, University of Illinois at Urbana-Champaign, Urbana, IL 61801, USA; E-Mail: spbrown1@illinois.edu; 4 Department of Forest Ecosystems and Environment, Oregon State University, Corvallis, OR 97331, USA; E-Mails: trappej@onid.orst.edu (J.M.T.); mycoroots@comcast.net (E.C.); 5 U.S. Forest Service, Forestry Sciences Laboratory, 3200 Jefferson Way, Corvallis, OR 97331, USA; 6 Department of Environmental Sciences, University of Helsinki, Lahti, FIN15140, Finland; E-Mail: rauni.strommer@helsinki.fi

**Keywords:** ectomycorrhiza, ECM, sporocarp, environmental DNA, Internal Transcribed Spacer, Large Subunit of the ribosomal RNA gene

## Abstract

Periglacial substrates exposed by retreating glaciers represent extreme and sensitive environments defined by a variety of abiotic stressors that challenge organismal establishment and survival. The simple communities often residing at these sites enable their analyses in depth. We utilized existing data and mined published sporocarp, morphotyped ectomycorrhizae (ECM), as well as environmental sequence data of internal transcribed spacer (ITS) and large subunit (LSU) regions of the ribosomal RNA gene to identify taxa that occur at a glacier forefront in the North Cascades Mountains in Washington State in the USA. The discrete data types consistently identified several common and widely distributed genera, perhaps best exemplified by *Inocybe* and *Laccaria*. Although we expected low diversity and richness, our environmental sequence data included 37 ITS and 26 LSU operational taxonomic units (OTUs) that likely form ECM. While environmental surveys of metabarcode markers detected large numbers of targeted ECM taxa, both the fruiting body and the morphotype datasets included genera that were undetected in either of the metabarcode datasets. These included hypogeous (*Hymenogaster*) and epigeous (*Lactarius*) taxa, some of which may produce large sporocarps but may possess small and/or spatially patchy genets. We highlight the importance of combining various data types to provide a comprehensive view of a fungal community, even in an environment assumed to host communities of low species richness and diversity.

## 1. Introduction

Many alpine glaciers have been retreating [1,2] since reaching a maximum during the Little Ice Age in the mid-1800s [3]. The substrates exposed by the retreating glaciers represent a challenging and extreme environment defined by a mineral substrate without organic legacies and minimal mineral nitrogen availability that limit ecosystem productivity [4]. In addition, the exposed deglaciated sites are burdened by extreme daily temperature oscillations combined with high irradiation, exposure to potentially damaging winds, and poor water retention [5,6]. These abiotic stressors challenge organismal establishment and survival in glacier foreland sites. However, such sites provide unique opportunities to explore community ecology in a setting that permits establishment of simple communities defined by low species richness [7,8].

Glacier forelands provide an elegant model system to study community assembly, facilitated by the low richness as well as the recently exposed denuded substrate. Jumpponen and Egerton-Warburton [9] described a mycorrhizal community assembly model derived from Diamond’s conceptual model based on environmental filtering [10]. This model posits that local and regional pools provide a transient propagule community upon which filtering acts and selects community members that are able to tolerate the prevailing abiotic conditions, maintain a competitive position in the community, and—importantly—find compatible hosts that the fungi colonize. Such models can be adapted and utilized to account for founder effects during early establishment as well as taxon physiological optima, environmental tolerances, and biotic interactions.

Mycorrhizal fungus communities can be surveyed in numerous ways. Sampling of sporocarps is expedient, but identification in previously unexplored areas or at sites with diverse communities may be laborious. Additionally, fruiting may be infrequent for some taxa and require repeated sampling, while some taxa form minuscule, resupinate sporocarps or fruit underground [11,12]. Mycorrhizal communities may also be assayed directly via analysis of mycorrhizae collected from the site [12,13,14] or even by sampling soils for direct sequencing of DNA metabarcode markers and screening the acquired data for mycorrhizal taxa [15,16]. These direct analyses have been greatly accommodated by the recent development of next generation sequencing (NGS) technology that has resulted in greater sequence yields at lower costs, making these technologies affordable to a variety of users [17]. With this decline of the cost of massively paralleled sequencing applications, direct metabarcode analyses might be the most efficient method to query the structure of resident communities.

Indeed, NGS technologies provide mycologists with tools to exponentially improve our appreciation of fungal communities in complex environments by a deeper and more comprehensive interrogation of environmental sequence data [18]. These tools have been rapidly adopted to expediently query both arbuscular mycorrhizal [19,20] and ECM communities [21,22]. However, these high throughput sequencing technologies are burdened with challenges of sequence quality, continuous development of best practices for analyses, and biases or errors that emerge from the use of these tools [18,23]. Further, it remains largely unclear whether different genomic regions provide similar views of the same community [24].

Here, we aimed to identify common mycorrhizal fungi that occur at a glacier forefront site that we have extensively studied over two decades [25]. We took advantage of existing datasets that utilized different methods and tools to evaluate the community assemblages and sought to pinpoint similarities and differences among them. We utilized existing sporocarp [26] and morphotyped ECM root tip data [27], as well as direct sequencing of nuclear metabarcode markers from field studies [23,28]. We aimed to synthesize these datasets to provide a comprehensive analysis of mycorrhizal communities assayed by different tools. In addition to comparing different research tools, we compared the variable internal transcribed spacer (ITS) region and its flanking large subunit (LSU) of the ribosomal rRNA gene. The limited mycorrhizal diversity at the site allows for near comprehensive assessment of the component taxa and minimizes stochastic impacts of incomplete sampling typical of studies in more diverse communities.

## 2. Results

### 2.1. Sporocarp Data for Ectomycorrhizal Fungi

Our previously reported sporocarp collections represented 13 species distributed across seven genera, sampled over the span of 17 collecting expeditions between 1988 and 1999 [26]. The recorded taxa included five *Cortinarius* ssp. (*C. decipiens*, *C. mutabilis*, *C. tenebricus*, plus two species that could not be identified beyond genus), *Hymenogaster glacialis*, *Inocybe lacera*, *Laccaria cf. montana*, *Lactarius uvidus*, an unidentified species of *Lactarius*, *Russula fragilis*, *Suillus aeruginascens* and *S. cavipes* [26]. *Hymenogaster glacialis* is hypogeous, *i.e*., truffle-like, fruiting belowground with spores dispersed by animal mycophagy [29]. The others are epigeous, fruiting as mushrooms aboveground and may be dispersed both by air currents and animal mycophagy. The two *Suillus* species are specific to *Larix* spp. *Larix lyallii*, subalpine larch, is infrequent in the Lyman foreland and occurs only at older positions in the chronosequence, but is rather common in the surrounding montane parkland communities [26].

**Figure 1:**
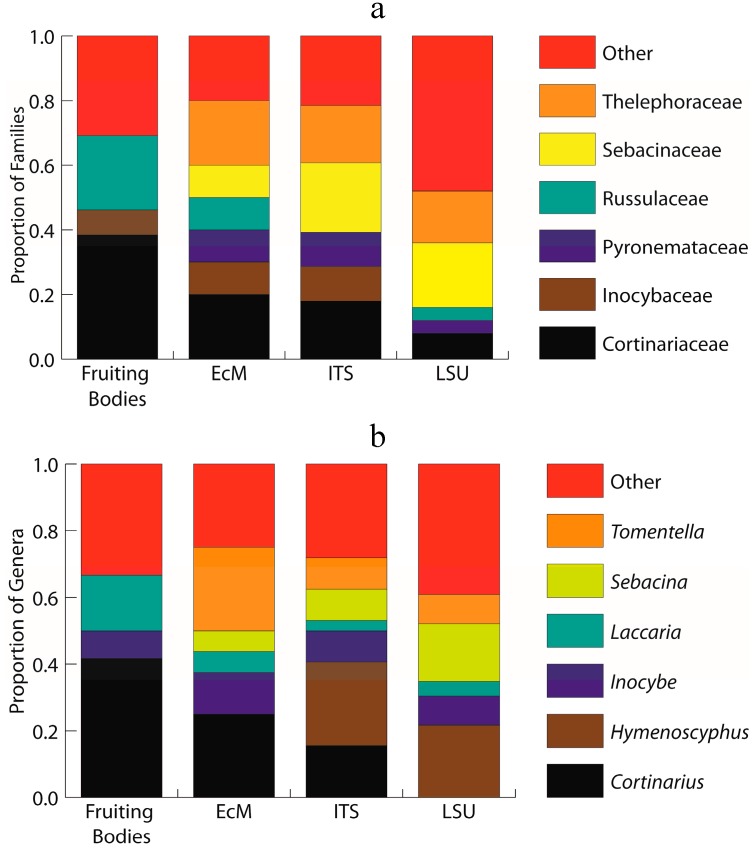
Stacked histogram representing the six most abundant ectomycorrhizal families (**a**) and genera (**b**) across the four datasets: sporocarp surveys (total of 7 families and 7 genera); morphotyped ectomycorrhizae (ECM; total of 7 families and 7 genera); environmental ITS metabarcode sequencing (ITS; total of 12 families and 15 genera); and, environmental LSU metabarcode sequencing (LSU; total of 12 families and 14 genera). Each bar is normalized to the total number within observed families (**a**) and genera (**b**) for each dataset.

The family that contains the genus *Cortinarius* (Cortinariaceae) is represented in the environmental sampling, as are families of *Inocybe* (Inocybaceae), *Laccaria* (Hydnangiaceae), *Lactarius* and *Russula* (Russulaceae), and *Suillus* (Suillaceae) (Figure 1a,b). The Hymenogastraceae, represented by *H. glacialis*, was never detected in the environmental morphotyped ECM or the metabarcode analyses.

Cázares and Trappe [29] conducted microscopic examination of scats and revealed that five of seven mammal species visiting this glacier forefront deposited spores of hypogeous ECM genera: *Cortinarius* (as *Thaxterogaster*), *Elaphomyces*, *Genea*, *Melanogaster*, *Rhizopogon*, and *Tuber*. In contrast, sporocarps of only one hypogeous fungus (*H. glacialis*) were found in the forefront, in that case under *Salix*. This new species was described by Cázares and Trappe [30] from that collection and has not been reported anywhere else since. The other hypogeous genera listed above were not present either as sporocarps or from environmental samples in the forefront. This is not surprising, inasmuch as mycophagy of hypogeous fungi by the animals involved was likely in conifer stands adjacent to the forefront, which they later visited. Spores of hypogeous fungi are almost all distributed by animals, which defecate the spores in their feces [31]. Consequently, spore deposition is stochastic rather than widely distributed and therefore the probability of their detection by a relatively small environmental sampling over a large landscape is low.

### 2.2. ITS Sequence Data for Morphotyped Ectomycorrhizae

**Figure 2:**
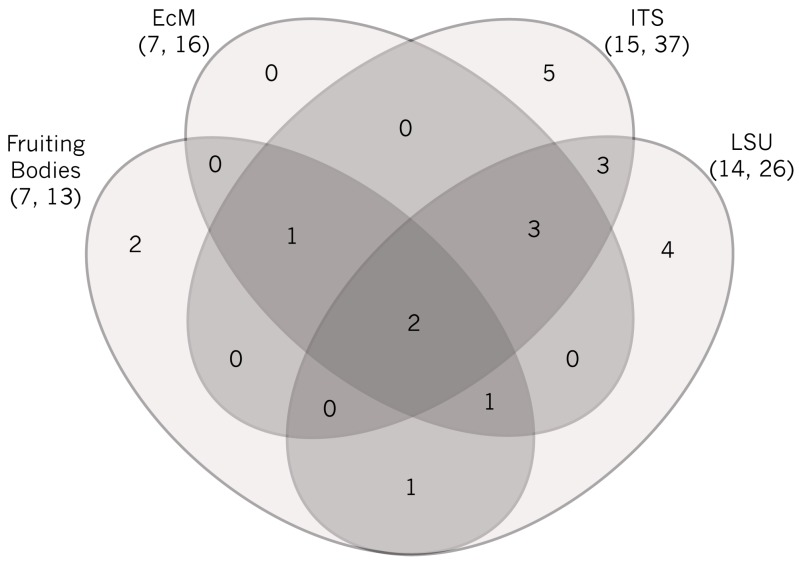
Four-way Venn diagram representation of shared and unique genera of ectomycorrhizal fungi across four datasets. Values within the diagram represent the number of genera. Note that direct comparison of numbers of species or operational taxonomic units is not possible. Total numbers of observed genera and species/OTUs are shown in parentheses. Only two genera were observed in all four datasets (*Laccaria*, *Inocybe*). Two genera were unique to the sporocarp dataset (*Hymenogaster, Lactarius*), six to ITS (*Rhizopogon*, *Hebeloma*, *Rhizoscyphus*, *Sistotrema*, *Thelephora*, *Wilcoxina*), and four to LSU (*Bankera*, *Hydnellum*, *Phallogaster*, *Tricholoma*), whereas none were unique to the morphotyped ECM dataset. The sporocarp dataset shared one genus with both the morphotyped ECM and ITS datasets (*Cortinarius*) that was absent in the LSU dataset, one that was only shared with the LSU (*Suillus*), and one that was shared with both the morphotyped ECM and LSU datasets (*Russula*) but absent in the ITS dataset. The morphotyped ECM, ITS and LSU datasets had three genera in common that were absent in the sporocarp dataset (*Peziza*, *Sebacina*, *Tomentella*), all of which produce small fruiting bodies that are likely to escape detection in sporocarp surveys. Finally, the two metabarcode datasets shared three genera that did not occur in any other dataset (*Hymenoscyphus*, *Piloderma*, *Trichophaea*). However, it is of note that the LSU dataset had other Thelephoraceae that could not be assigned to a genus using the available data/databases.

Our morphotyped ECM data included 26 accessioned reads and represented five orders (Agaricales, Pezizales, Russulales, Sebacinales and Thelephorales) that commonly have species that form ECM. Two additional Orders (Chaetothyriales and Sordariales) that rarely include ECM-forming species were observed; we opted to exclude them as ECM taxa here. The data represented a total of twelve genera, seven of which are likely mycorrhizal (Figure 1b; *Cortinarius*, *Inocybe*, *Laccaria*, *Peziza*, *Russula*, *Sebacina*, and *Tomentella*). Some sequences could only be assigned to an order (unclassified Sordariales) or a family (Hydnangiaceae, Pyronemataceae, Sebacinaceae). Two additional sequences in this dataset represent taxa that are unlikely to form mycorrhizae (*Exophiala*, *Podospora*). The Naïve Bayesian Classifier combined with the UNITE INSD mostly provided a high bootstrap support for these assignments, albeit rarely at the subgeneric levels (Supplemental Table S1). As a result, we confined our description of the results to the generic levels (Figure 1b; Figure 2).

### 2.3. Environmental ITS Amplicon Data

The environmental sequence data of 463 non-singleton ITS OTUs derived from 454-pyrosequencing represented a more diverse fungal community at the glacier forefront study site. However, sequences assigned to mycorrhizal taxa were infrequent and comprised a small proportion (12.9%) of all sequence data and only 8.0% of all OTUs clustered at 97% similarity. Interestingly, we detected no sequences assigned to Phylum Glomeromycota in the ITS dataset, although a fifth of the samples originated from rhizospheres of the arbuscular mycorrhizal *Luetkea pectinata*, which has that type of mycorrhiza in this glacier forefront [7].

Taxomically speaking, the sequence data were assigned to a total of 55 orders with putative ECM taxa distributed across eight orders (Agaricales, Atheliales, Boletales, Cantharellales, Helotiales, Pezizales, Sebacinales, Thelephorales), an additional eight could not be assigned to orders, and the remaining 39 represented various ecologies including common soil inhabiting taxa (e.g., Archaeorhizomycetales, Mucorales, Mortierellales), entomopathogens (Entomophthorales), common saprobes (e.g., Eurotiales, Sordariales), plant pathogens (Ustilaginales), and lichenicolous fungi (Lecanorales). A total of 37 OTUs were assigned to a total of fifteen genera that likely form ECM (Figure 1b; *Cortinarius*, *Hebeloma*, *Hymenoscyphus*, *Inocybe*, *Laccaria*, *Peziza*, *Piloderma*, *Rhizopogon*, *Rhizoscyphus*, *Sebacina*, *Sistotrema*, *Thelephora*, *Tomentella*, *Trichophaea*, and *Wilcoxina*); two possible ECM OTUs could only be assigned to the level of a family (Sebacinaceae and Thelephoraceae). The bootstrap support for these generic assignments varied from 100% for some taxa (*Cortinarius*, *Hebeloma*, *Inocybe*) to as low or less than 10% for others (*Sebacina*) (Supplemental Table S2). These results likely indicate uneven database representation for the taxa detected at our study site.

### 2.4. Environmental LSU Amplicon Data

In addition to the environmental ITS data, we analyzed 517 non-singleton LSU OTUs that included some representatives of arbuscular mycorrhizal fungi that are rarely represented in the environmental ITS datasets. Similar to the ITS data, mycorrhizal sequences were infrequent: sequences that represented arbuscular mycorrhizal taxa comprised only 0.01% of all analyzed sequences and 0.8% of all non-singleton OTUs; sequences that represented ECM taxa comprised 9.2% of all sequences and 4.8% of all OTUs.

These LSU data included representatives of taxa from two Glomeromycotan orders (Diversisporales, Paraglomerales) and nine orders with putative ECM (Agaricales, Atheliales, Boletales, Helotiales, Hysterangiales, Pezizales, Russulales, Sebacinales, and Thelephorales). Four OTUs were assigned to four arbuscular mycorrhizal genera (*Acaulospora*, *Entrophospora*, *Pacispora*, and *Paraglomus*). The LSU data included a diverse assembly of 26 OTUs assigned to thirteen potentially ECM-forming genera (Figure 1b; *Bankera*, *Hydnellum*, *Hymenoscyphus*, *Inocybe*, *Laccaria*, *Peziza*, *Phallogaster*, *Piloderma*, *Russula*, *Sebacina*, *Suillus*, *Tomentella*, *Tricholoma* and *Trichophaea*) or two families (Sebacinaceae, Thelephoraceae). Similar to the environmental ITS dataset, the bootstrap support for these assignments was variable and included taxa with support as high as 100% (*Acaulospora, Sebacina*), as well as many with support as low as <30% [*Bankera* (bootstrap support = 26), *Entrophospora* (bootstrap support = 11), *Hymenoscyphus* (bootstrap support = 23), *Pacispora* (bootstrap support = 10)] (Supplemental Table S3).

## 3. Discussion

We mined previously published sporocarp and morphotyped ECM as well as environmental ITS and LSU metabarcode data to identify taxa that occur at our glacier forefront study site and likely represent organisms that occur in a deglaciated environment. Multiple environmental stressors challenge organismal persistence in this system [4,32]: the absence of organic legacies and minimal supply of nitrogen; extreme diurnal temperature fluctuations; few barriers against wind damage; poor water retention; and intense irradiation. Further, the glacier forefront sites are often at high latitudes or altitudes and have short growing seasons and heavy snow during winter. The combination of these abiotic stressors makes the glacier forefronts biologically challenging for establishment and survival.

Most ECM genera in Lyman Glacier are widely distributed and circumpolar in (sub-)alpine and (sub-)arctic habitats [33]. *Inocybe lacera* exemplifies species of wide range and habitat diversity, particularly those disturbed by glaciation, earth movements, or anthropogenic activities. To name a few records, *I. lacera* has been observed in glacier forefronts from the North Cascade Mountains of Washington State north to Alaska’s Kenai Peninsula [34], east to subalpine zones of the Rocky Mountains [35], Arctic Canada, Greenland, Svalbard, Lapland, and the Alps to eastern Siberia [36], thence to early successional communities on Japan’s Mt. Fuji [37].

Although inadequate to produce a complete list of ECM genera or species that produce macroscopic sporocarps at our site, our data included representatives of seven genera. Six of these belong to genera detected in the environmental samples and four to genera observed among the morphotyped ECM. The seventh, *Hymenogaster glacialis*, was hypogeous and found only once, under stones during sampling soil under a shrub willow [30]. While our environmental sequence datasets included a large number of OTUs (37 ITS and 26 LSU) that likely form ECM, we highlight the importance of combining various data types to provide a comprehensive view of a community—even if perceived to be low in organismal diversity and richness. Use of sporocarp detection may be particularly important for hypogeous taxa; their spore deposition is stochastic and their detection probability low in limited, small volume environmental samplings across large landscape units.

The morphotyped ECM and fruiting body datasets were largely complementary and included—in addition to shared genera (*Cortinarius*, *Inocybe, Laccaria*)—several that occurred in one but not another survey (*Hymenogaster*, *Lactarius* and *Suillus* in the fruiting body collections; *Sebacina* and *Tomentella* in the ECM dataset). Clearly, the small and ephemeral sporocarps of *Sebacina* and *Tomentella* are likely to be missed in fruiting body surveys. The lack of detection of the taxa with large sporocarps, e.g., *Suillus aeruginascens* and *S. cavipes*, present in the sporocarp dataset, may be attributable largely to their ECM specificity to the infrequent *Larix lyallii* hosts in the forefront as well as small genet sizes in the soil.

The capacity to molecularly detect ECM far exceeds that of casual sporocarp surveys. Further, the morphotyped ECM dataset included no genera that were only observed in that dataset, although it did include one that remained undetected in the ITS metabarcode dataset (*Russula*) and one that was undetected in the LSU metabarcode dataset (*Cortinarius*). In contrast, the sporocarp dataset included ECM genera that remained undetected in the morphotype sequencing or in the environmental ITS or LSU metabarcode datasets. For example, no *Russula* or *Suillus* were observed in the ITS dataset, whilst they were included in the sporocarp dataset. Further, it is notable that although both the fruiting body and morphotyped ECM datasets detected *Cortinarius* and *Inocybe*, no *Cortinarius* was observed in the LSU dataset. In addition, none of the molecular datasets detected the two *Lactarius* spp. reported to fruit adjacent to the terminal moraine or the *Hymenogaster* reported as a single occurrence at the study site [26,30]. There are several potential explanations for these observations and it is important to bear in mind that the different assessment tools were never employed simultaneously and that the occurrence of fungal taxa and genotypes is spatially and temporally heterogeneous. Among the additional factors are the primer, extraction, and sequencing biases that prohibit the comprehensive taxon evaluation in any study as well as the small sample volume included in the molecular surveys.

Direct sequencing of morphotyped ECM has provided a wealth of information for a number of systems and communities [12]. Although our data were derived from ECM that differed in gross morphology [27], they still included taxa that unlikely form ECM (*Exophiala*, *Podospora*) but rather represent casual root-inhabiting fungi. Similar to these observations, the environmental ITS and LSU metabarcode data that broadly sampled soil-inhabiting communities included large proportions of non-mycorrhizal taxa. NGS datasets often include non-target organisms, even when aiming to explicitly focus on symbiotic communities. For example, recent studies that sampled ECM roots included large numbers of OTUs that represent root-associated pathogens, endophytes, and simply soil fungi that occupy the soil-ectomycorrhizosphere interface [13,14]. Such observations are not unique, although the proportion of ECM OTUs in our ITS and LSU datasets is lower than those in surveys of temperate established forest soils. Voříšková and collaborators [38] observed ca. 20% ECM OTUs in the surface topmost L horizon in oak forest soils and ca. 65% ECM OTUs in the Ah horizon soils. Similarly, Coince *et al*. [15] and Oliver *et al*. [16] reported that less than half of the observed OTUs represented known or putative ECM taxa in temperate forest soils. The difference between our data from a primary successional glacier forefront and those from others describing fungal communities in established temperate forests is likely attributable to a lesser ECM plant cover plus the low ECM hyphal density in the early successional soils of the glacier forefront site.

The environmental datasets targeting two different regions of the ribosomal RNA gene repeat yielded large numbers of OTUs from soil samples collected at the glacier forefront: a total of 463 non-singleton ITS and 517 non-singleton LSU OTUs derived from 454-pyrosequencing. These data represented diverse fungal communities with 37 ITS and 25 LSU OTUs that may form ECM. Yet, the numbers of detected genera resemble those observed in the fruiting body and ECM morphotype datasets: the ITS OTUs were distributed across fifteen and the LSU OTUs across fourteen ECM genera. Some of the genera that we included may or may not form ECM (e.g., *Hymenoscyphus*), although there is limited evidence that these genera may include ECM members [39]. Similarly, some taxon assignments received only poor bootstrap support (e.g., *Entrophospora*, *Pacispora*, *Sebacina*), and some may represent taxa that are unlikely to occur in the study area (e.g., *Phallogaster*). Both datasets also included some OTUs that could only be assigned to higher taxonomic ranks. Unfortunately, these datasets cannot be directly compared—we must rely on comparisons at a genus level. While primer biases and different database population densities likely explain some of the observed differences, there are also considerable overlaps between the two datasets. Both analyses consistently detected OTUs assigned to *Hymenoscyphus*, *Inocybe*, *Laccaria*, *Peziza*, *Sebacina*, *Tomentella*, and *Trichophaea*. Many of these also overlap with both fruiting body and ECM surveys (e.g., *Inocybe* and *Laccaria*). Perhaps a reasonable conclusion is that no dataset alone comprehensively characterizes the ECM communities—even in a relatively simple system—but complementary approaches that combine the easy detection of large sporocarps and the power of deep sequencing afforded by the NGS analyses might result in the most comprehensive taxon lists.

As many of the fungi observed in the metabarcoding datasets do not form ECM, many of those detected in soil may never assume metabolic activity, but be present only as a dormant and inactive propagule pool or as residual naked DNA detectable via PCR-based approaches [40,41]. The DNA coding for the ribosomal RNA (rRNA) genes or their spacer regions have proven extremely useful for environmental sampling of ECM [12]. The environmental rRNA can serve as a viable alternative to specifically detect active communities [42,43]; rRNA is unstable and thus the metabolically active organisms will be preferentially targeted with a greater temporal resolution than that afforded by the analysis of environmental DNA [41]. However, the analyses of rRNA are not without challenges. Transport and preservation of the RNA acquired from remote field sites, such as the glacier forefront described here, and high fidelity transcription using transcriptases with optimal activities below annealing temperatures for taxon/locus specific primers can prove problematic. Surprisingly, the expectation that the RNA-derived view of the community would be a subset of that observed through analyses of DNA often proves erroneous and the RNA- and DNA-derived communities tend to be distinct [44]. Targeting ECM fungi in remote, difficult to access field sites using rRNA and its transcription are perhaps not optimal compared with the expedience and ease of analyses of more stable DNA.

Most of the data included here focused on the ECM fungi. Yet, our LSU data included OTUs that were assigned to Glomeromycota, thus indicating the establishment of arbuscular mycorrhizal fungal communities at our glacier forefront site. The Glomeromycotan sequences were few and represented only four OTUs. Although it may be tempting to conclude that the low detection rate represents their low occurrence, one must bear in mind that arbuscular mycorrhizal fungi often constitute only a small proportion of sequences and taxa in environmental NGS analyses. Our earlier studies show that the most recently exposed substrates rarely support arbuscular mycorrhizal plants (see [7,25]). These observations are similar to those of Reeves *et al.* [45], who reported that plants establishing in systems with no plant cover are often nonmycorrhizal, whereas those that dominate the later successional stages are mycorrhizal. Arbuscular mycorrhizal fungi tend to increase over time in many early successional ecosystems, suggesting their importance in facilitating plant establishment and survival under harsh conditions that define many early successional communities [46]. After establishment, the mycorrhizal hosts may produce soil inoculum, thus facilitating the establishment of other mycorrhizal plants [47]. Our earlier root studies showed arbuscular mycorrhizae on plants in the older parts of the chronosequence, but Glomeromycotan spores are infrequent [7]. While the sampling for Glomeromycotan fungi is perhaps only coincidental with the analyses of the broader soil inhabiting fungi, their presence in our LSU dataset unequivocally confirmed arbuscular mycorrhizal communities in this glacier forefront. Many of the ECM fungi may arrive aerially, but dispersal of Glomeromycota—similar to the hypogeous ECM taxa—relies on the movement of spore-containing soil by landslides, erosion, or animal vectors (likely on the feet or in the scats of visiting animals; see also [48]). Although animal vectors are perhaps the most frequent, avalanches and landslides at the forefront borders may further contribute to propagule arrival. The low colonization and infrequent detection of Glomeromycotan spores strongly suggest spore dispersal limitation and/or abiotic environmental filtering of hosts and root-inhabiting symbionts in these habitats.

What do these data tell us about the fungal communities in a biologically challenging and sensitive environment exemplified by the glacier forefront? It is possible that the most important messages are between the lines. The detection of different taxa in each of the datasets, in addition to the need for complementary surveys, highlights the site heterogeneity as well as the often fundamentally different views of the resident fungal communities that the different survey methods provide. While extreme environments are commonly considered relatively homogeneous, we emphasize the environmental heterogeneity that may result from microtopological differences as exemplified by “safe sites” that facilitate plant establishment [5] or from environmental modulation by establishing plants [27]. The stress gradient hypothesis [49] highlights the increasing importance of the positive, facilitative interactions with increasing stress. We posit that during early ecosystem development, these positive interactions indeed facilitate the community assembly, *i.e.*, the establishment of symbiotic associations is mandatory for maintaining the plant communities and their fungal symbionts. In contrast, later during ecosystem development, within-guild competitive and antagonistic interactions likely increase in importance. Issues that remain open beyond generating lists of occurring taxa include the temporal and spatial heterogeneity of symbiotic communities, longevity of genets, and importance of founder effects in these systems. Clearly, well-informed and ambitious manipulative or observational studies are necessary to further elucidate the community dynamics in biologically demanding environments.

We also wish to sound an alarm of caution. While temporal and spatial heterogeneity undoubtedly contribute to the observed differences among the datasets that we mined here, it is also important to highlight the risks of too far-fetching comparisons among independent studies that attempt to evaluate similar research questions using different tools. Our datasets and analyses serve as an example in many ways. First, our data highlight the differences between sporocarp and molecular surveys. While the former may be efficient in detecting large fruiting bodies emerging from potentially small and discrete ramets, the latter detects the dominants that comprise a substantial proportion of a symbiotic mycorrhizal community. Second, although the expedient deployment of NGS tools is tempting, sampling strategies, analyzed regions, and database annotations deserve serious thought. Our data show that the NGS analyses, despite the large volume of data they may produce, failed to detect taxa observed in the sporocarp surveys or ECM morphotyping studies. Third, the two analyzed NGS datasets included a large number of genera that were present in only one of those datasets. Clearly, there are several underlying reasons for these observed differences and our data do not permit the elucidation of them. However, we wish to draw attention to the potential issues of primer biases and/or PCR biases that may result from length polymorphisms. Finally, the database coverage as well as annotation accuracy are of crucial importance, especially if direct sequence comparisons are impossible and one must rely on comparisons of taxa whose affinities have been derived from different datasets.

## 4. Materials and Methods

### 4.1. Study Site

Lyman Glacier (ca. 1800 m a.s.l.) in the Glacier Peak wilderness area is located in the North Cascade Mountains (48° 10′ 52″ N, 120° 53′ 87″ W Washington, DC, USA). The glacier has receded steadily since the 1890s, opening a 1000-m long forefront to pioneering plants and fungi [50]. Our previous contributions describe the environmental attributes of this site [25] that led to its selection among the >700 glaciers [51] in the North Cascades Mountains. Similar to many other glacier forefronts, the substrate texture and chemistry at this site are heterogeneous, representing differences in the bedrock deposited as outwash and moraines.

### 4.2. Sporocarp Data

Fungal fruiting tends to be ephemeral or patchy and the sporocarps often short-lived. To account for the unpredictable fruiting and the short detection timeframe, we visited the remote study site 17 times over 12 years as described earlier [26]. The surveys covered the entire glacier forefront length (~1000 m) and concentrated on the central area with a rather homogeneous substrate and confined by glacial melt water streams and steep slopes [50]. Sporocarps representing 13 species and 7 genera were sampled in the course of the expeditions between 1988 and 1999. Lacking electricity or gas-burning facilities for drying specimens, we dried them in the sun, weather permitting, or on a stack of screens suspended over a campfire. When the field program began, molecular methods were not commonly in use, so attention was focused on preservation of morphological characters. These were adequate for identification and the collections were identified by standard microscopic measurements coupled with the macroscopic data recorded for fresh characters. However, we later discovered that the primitive drying methods precluded survival of useable DNA [27] and we have failed to obtain visible PCR amplicons from the sporocarps [27]. Accordingly, the sporocarp data could not be included in further sequencing analyses.

In addition to the sporocarp collections, Cázares and Trappe [29] collected scats of mammal mycophagists for examination of spores of fungi that had been grazed. Some off these animals lived at the edges or in the older, more vegetated parts of the forefronts (American pikas, hoary marmots, yellow pine chipmunks), but tracks indicate that they also frequent the more exposed areas with lesser vegetation. In addition to the small mycophagists, some larger mycophagal mammals, including mule deer and mountain goats, visited the site and contributed fecal pellets but did not reside in the forefront. The presence of hypogeous and occasional epigeous fungi was determined by spore sizes, shapes and ornamentation [52].

### 4.3. Morphotyped Ectomycorrhizae Sequence Data

Mycorrhizas from 30 commonly occurring shrub willows representing the commonly occurring taxa (*Salix commutata* and *S. planifolia*) were sampled and analyzed as reported in Trowbridge and Jumpponen [27]. That study screened ECM via PCR-amplification of the ITS region and restriction enzyme digestion with AluI and HinfIII [53,54], resulting in the identification of distinct Restriction Fragment Length Polymorphism (RFLP) phenotypes that were subsequently accessioned to GenBank (accessions AY187593-AY187616). We harvested these sequence data to obtain a view of the ECM data and acquired a total of 26 accessions of morphotyped mycorrhizas that were sequenced for ITS (resultant .fasta file is available as a Supplementary File S1). These sequence data were assigned to taxa using the Naïve Bayesian Classifier [55] as implemented in MOTHUR [56] (50% bootstrap support threshold) against the UNITE-curated International Nucleotide Sequence Database (INSD) reference database [57]. Complete taxonomic strings are available as Supplementary Table S1.

### 4.4. Environmental ITS Amplicon Data

We harvested raw sequence data from Brown and Jumpponen [28] (SRA BioProject accession PRJNA201483). Briefly, rhizosphere soils from 4 plant species with different mycorrhizal ecologies (*Abies lasiocarpa* (Hook.) [ectomycorrhizal], *Luetkea pectinata* Kuntze [arbuscular mycorrhizal], *Phyllodoce empetriformis* D.Don [ericoid mycorrhizal], and *Saxifraga ferruginea* Graham [non-mycorrhizal]) as well as non-vegetated soils were collected along the Lyman Glacier forefront. Amplicons of the ITS1, 5.8S, and ITS2 regions were generated with fungal specific primers ITS1f [53] and ITS4 [58] and 454-pyrosequenced using FLX Titanium chemistry as described in Brown and Jumpponen [28]. The ITS sequence data were quality controlled and processed using the program MOTHUR (v.1.33.3, [56]; see also [24]) and included a total of 53,012 sequences after quality control. Sequences were assigned to 819 Operational Taxonomic Units (OTUs) at 97% sequence similarity (UPGMA) and 356 singleton sequences (sequences that occur only once in the dataset) were removed as they may represent PCR and/or sequencing artifacts [23]. Representative sequences of 463 non-singleton ITS OTUs were randomly selected (resultant .fasta file is available as Supplementary File S2) and assigned to taxa using the Naïve Bayesian Classifier [55] as implemented in MOTHUR [56] (50% bootstrap support threshold) against the UNITE-curated International Nucleotide Sequence Database (INSD) reference database [57]. Each OTU was assigned to a putative ecology (mycorrhizal/other) based on genus level affinities. The taxon assignment of the OTU representative sequences is available as a Supplementary Table S2.

### 4.5. Environmental LSU Amplicon Data

To expand our potential inference, we included an additional dataset representing the large subunit (LSU) of the ribosomal RNA gene (SRA BioProject accession PRJNA201483). These data are described in a previous publication [24]. Briefly, LSU amplicons were generated targeting the variable regions D1 and D2 using the primers LR0R [59] and LR3 [60] from the same DNA extracts sampled for the ITS data (above) and 454-pyrosequenced with FLX Titanium chemistry. Similar to the ITS datasets, the LSU data were quality controlled [24] and aligned against a modified James *et al*. [61] LSU reference alignment. The sequences were clustered to 911 OTUs (including 394 singleton OTUs that were culled as potential artifacts [23]) at 97% sequence similarity (UPGMA). Representative sequences for each of the 517 non-singleton OTU (.fasta file is available as Supplementary File S3) were sampled and assigned to taxa using the Naïve Bayesian Classifier [55] and the Ribosomal Database Project 28S training dataset (v.7) [62]. We use the taxon annotations that are congruent with the available reference databases and some may consequently differ from current nomenclature. The taxon assignment of the representative sequences is available as a Supplementary Table S3.

## 5. Conclusions

We summarized four different datasets used to characterize mycorrhizal communities in a biologically challenging glacier forefront ecosystem. While we focused mainly on ECM fungi, few detected environmental sequences assigned to Glomeromycota confirm their presence in our glacier forefront system. The NGS analyses yielded overwhelmingly the most species rich views of the ECM community compared with analyses of sporocarps or morphotyped ECM. Despite the comparatively high sequencing depth—especially given that the glacier forefront system was characterized by low species diversity, our data did not capture many genera observed in more traditional sporocarp surveys. While the reasons for the observed differences remain unclear, a combination of experimental approaches, including fruiting body surveys to capture large sporocarps that may represent small genets, direct analyses of mycorrhizal communities, and deep NGS analyses are likely to produce the most comprehensive views of these communities. It is also important to bear in mind that sequence library preparation and database annotations play a crucial role in the accuracy of comparisons made between molecular and morphology data.

Our analyses identified many ECM and some arbuscular mycorrhizal genera that occur at this glacier site. Taxa that occurred in most—if not all—datasets included many that often form small sporocarps (*Cortinarius*, *Inocybe*, *Laccaria*) or some that produce sporocarps that commonly escape visual detection (*Thelephora*, *Tomentella*, *Sebacina*). While it may be self-evident that few mycorrhizal fungi remain metabolically active in the absence of their hosts, our understanding of the controls of the community assembly and community successional dynamics in challenging environments is rudimentary at best.

## Supplementary Files

Supplementary File 1
